# Home Tobacco Use Policies and Exposure to Secondhand Tobacco Smoke: Findings from Waves 1 through 4 of the Population Assessment of Tobacco and Health (PATH) Study

**DOI:** 10.3390/ijerph18189719

**Published:** 2021-09-15

**Authors:** Cheryl Rivard, Anthony Brown, Karin Kasza, Maansi Bansal-Travers, Andrew Hyland

**Affiliations:** Cheryl Rivard, Roswell Park Comprehensive Cancer Center, Elm & Carlton Streets, Buffalo, NY 14263, USA; Anthony.Brown@RoswellPark.org (A.B.); Karin.Kasza@RoswellPark.org (K.K.); Maansi.Travers@RoswellPark.org (M.B.-T.); Andrew.Hyland@RoswellPark.org (A.H.)

**Keywords:** secondhand smoke, home, policy

## Abstract

Background: The 2006 Surgeon General’s Report recommended the elimination of smoking in homes as an effective protective measure against the harmful effects of secondhand tobacco smoke exposure. This study aims to examine trends in the prevalence and levels of the adoption of home tobacco use policies specifically for cigarettes, e-cigarettes, smokeless tobacco, and the relationships between home tobacco use policies and self-reported exposure to secondhand tobacco smoke. Methods: This study utilizes data from Wave 1 (2013–2014) through Wave 4 (2016–2018) of the Population Assessment of Tobacco and Health (PATH) Study, a large prospective cohort study of youths and adults in the United States which collected information about both smoke-free and tobacco-free home policies. We present the weighted, population-based, self-reported prevalence of home tobacco use policies overall and by product, and the average number of self-reported hours of secondhand smoke (SHS) exposure by levels of home tobacco policy and by survey wave. In addition, we examine the characteristics of those who adopted (by yes or no) a home tobacco use ban between survey waves. Results: We found a high prevalence of completely tobacco-free home policies (69.5%). However, 10.6% of adults allow the use of any type of tobacco product inside their homes, and 19.8% have a policy allowing the use of some types of tobacco products and banning the use of others. Adults with a complete tobacco use ban inside their homes were more likely to be nonusers of tobacco (79.9%); living with children in the home (71.8%); at or above the poverty level (70.8%); non-white (76.0%); Hispanic (82.7%); and aged 45 or older (71.9%). The adoption of 100% tobacco-free home policies is associated with a 64% decrease in secondhand smoke exposure among youths and a 69% decrease in exposure among adults. Conclusions: Most US adults have implemented tobacco-free home policies; however, there is still exposure to SHS in the home, for both adults and children, particularly in the homes of tobacco users. Additional research should investigate tobacco-free home policies for different types of products and what effect they have on future tobacco use behaviors.

## 1. Introduction

Cigarette smoking remains the leading cause of preventable death in the United States, and the dangers of exposure to secondhand smoke (SHS) to health have been confirmed [1,2]. The 2006 Surgeon General’s Report recommends the elimination of smoking in homes as an effective protective measure against the harmful effects of secondhand tobacco smoke exposure [3]. While the exposure to SHS has significantly decreased for adults in the US due to the increased institution of smoke-free workplace policies, with 61% of the US population covered by 100% smoke-free state or local workplace laws [4], both adults and children continue to be exposed in their home environments [5].

The tobacco product landscape has expanded over the past several years to include various alternative products, such as electronic nicotine delivery systems (ENDS; e.g., vape pens, e-cigarettes) [6] and smokeless tobacco products such as snus (a Swedish-style moist snuff tobacco) [7]. In contrast to combustible tobacco products, e-cigarettes do not produce sidestream emissions. Nevertheless, secondhand aerosol exposure from ENDS can expose nonusers, including children, to harmful and potentially harmful constituents, such as nicotine, ultrafine particulates, and volatile organic compounds, among others [8]. For smokeless tobacco products, the issue is different. Smokeless tobacco has even been marketed as a substitute product safe to use in smoke-free environments [9]. Even so, some research has identified that allowing the use of tobacco products in the home is independently associated with a greater initiation of tobacco products among young people in the home [9] and may decrease tobacco cessation efforts [10].

Most adults believe that SHS is dangerous for nonsmokers [11], and these risk perceptions are associated with the voluntary adoption of smoke-free home policies [12]. In addition, comprehensive tobacco control policies, including strong public and smoke-free workplace laws, appear to effectively increase the prevalence of smoke-free home policies, thereby protecting all residents, including children, from the harmful effects of SHS [13,14]. However, few studies have examined home policies on the use of ENDS or smokeless tobacco. Prohibiting the use of ENDS in the home is less common with users of these products [15,16]. Additionally, a recent examination of parental interview data from pediatric practices found that parent dual users were more likely to have smoke-free than vape-free home policies, suggesting that some may perceive e-cigarette aerosols as safe for indoor use [17]. Rules about tobacco use inside the home are associated with lower levels of youth initiation of smokeless tobacco use [18,19]. However, none of these studies examined trends in adopting these policies over time or compared levels of SHS exposure across different home tobacco use policy levels.

The present study aims to fill in the gaps in the literature concerning home policies about the use of different types of tobacco products by examining trends in these policies over time and their association with SHS exposure. This study utilizes data from the Population Assessment of Tobacco and Health (PATH) Study, a large prospective cohort study of youths and adults in the United States which collected information about smoke-free and tobacco-free home policies.

## 2. Methods

The PATH Study is a nationally representative, longitudinal cohort of 45,971 adults and youths aged 12 years and older in the US. The study is a collaboration of the National Institutes of Health (NIH), through the National Institute on Drug Abuse, with the Food and Drug Administration’s Center for Tobacco Products, along with contracted work performed by Westat. Using computer-assisted audio self-interviews, information was collected on behaviors, beliefs, and health outcomes related to tobacco products and their use. The current analysis uses the PATH Study data from Wave 1 (collected from September 2013 to December 2014); Wave 2 (October 2014 to October 2015); Wave 3 (October 2015 to October 2016); and Wave 4 (December 2016 to January 2018) to assess home tobacco use policies and SHS exposure.

A four-stage stratified area probability sample design was used in Wave 1 of the PATH Study to select adults and youths aged 12 to 17 from the US civilian, non-institutionalized population (CNP). The Wave 1 weighted response rates were 54.0 percent for the household screener and 74.0 percent for the adult interview. Population and replicate weights were constructed to compensate for variable probabilities of selection, differential non-response rates, and possible deficiencies in the sampling frame (e.g., under-coverage of certain population groups). Combined with the probability sample, the weights allow analyses to compute robust estimates and represent the non-institutionalized, civilian US population aged 12 and older. The PATH Study design and methods are published elsewhere [20]. Details on the survey interview procedures, questionnaires, sampling, weighting, and accessing the data are available at https://doi.org/10.3886/ICPSR36498.v6 (accessed on 27 August 2021). The study was conducted by Westat and approved by the Westat Institutional Review Board. All participants aged 18+ provided informed consent, while youths aged 12–17 provided assent with parental consent.

## 3. Measures

Home Tobacco Use Policies. Home tobacco use policies were assessed using three distinct measures. First, participants were asked, “For tobacco products that are burned, such as cigarettes, cigars, pipes, or hookah, which statement best describes the rules about smoking a tobacco product inside your home?” Second, participants were asked, “Now think about e-cigarettes and other electronic nicotine products. Which statement best describes the rules about using these products inside your home?” Lastly, they were asked, “Now think about other tobacco products that are not burned, like smokeless tobacco and dissolvable tobacco. Which statement best describes the rules about using these products.

Inside your home?” The response options for the three items were: (a) “It is not allowed anywhere or at any time inside my home”; (b) “It is allowed in some places or at some times inside my home”; or (c) “It is allowed anywhere and at any time inside my home.” These responses were then used to create a derived variable describing tobacco home policy status with four levels (no tobacco product use banned; combustible tobacco products banned but smokeless or ENDS use allowed; smokeless or ENDS use banned but combustible tobacco allowed; or all three product types banned).

*Home Tobacco Use Policy Adoption.* Respondents were classified as having adopted a home tobacco use policy if they reported that all tobacco product types were allowed inside their homes at Wave 3 and reported that at least 1 type of tobacco product was banned (not allowed anywhere or at any time) in their homes at Wave 4.

*Tobacco Use.* Past 30-day tobacco use behaviors were assessed. Products were grouped into combustible products, including conventional cigarettes, cigars, cigarillos, filtered cigars, hookahs, pipe tobacco, or non-combustible products, including ENDS, loose snus, snus pouches, moist snuff, dip, chewing tobacco, spit, and dissolvable tobacco. Responses were used to classify participants as either (1) no past 30-day tobacco use; (2) past 30-day use of combustible tobacco only; (3) past 30-day use of ENDS or smokeless tobacco only; or (4) past 30-day poly-use of combustible, ENDS, and/or smokeless tobacco.

*Secondhand smoke exposure.* SHS exposure was measured by asking, “During the past seven days, about how many hours were you around others who were smoking [whether or not you were smoking yourself]? Include time in your home, in a car, at school, or outdoors.”

*Other measures.* Other measures included in the analyses are children in the home (yes or no); poverty status (below poverty level, at or above poverty level); sex (male, female); race (white only, black only, other); ethnicity (Hispanic, not Hispanic); and age group (18–24, 25–44, 45+).

## 4. Analysis Plan

We conducted analyses in Stata/SE version 14.1 using survey procedures to account for weighting. We present the weighted, population-based, prevalence of home tobacco use policies using the balanced repeated replication (BRR) method with Fay’s adjustment set to 0.3, using cross-sectional weights. We examined the characteristics of adults with home tobacco use bans at Wave 4 using chi-square tests. We examined the average number of hours of SHS exposure by the change in home tobacco policy and by survey wave, using longitudinal (all-waves) weights. Logistic regression models were constructed using Wave 4 longitudinal, all-waves weights to evaluate the characteristics of those who adopted (by yes or no) varying levels of home tobacco use bans between Waves 3 and 4.

## 5. Results

### 5.1. Home Tobacco Use Policy Status

The varying levels of home tobacco use policies among adults at Wave 4 are presented in Table 1. Fourteen point five percent completely ban combustible tobacco products while allowing the use of smokeless tobacco or ENDS. However, only 5.3% completely ban smokeless tobacco or vaping while allowing combustible tobacco product use. Sixty-nine point five percent of adults do not allow the use of any of the three types of tobacco products in their homes, and 10.6% have no ban on any of the three types of tobacco products. Among tobacco nonusers (no past 30-day tobacco use), the prevalence of a home tobacco use policy banning the use of all products was 79.9%, compared to 47.0% among users of combustible tobacco only, 28.9% among users of ENDS or smokeless tobacco, and 33.1% among poly-users (combustible tobacco and smokeless tobacco and/or ENDS). Adults with a complete tobacco use ban inside their homes were more likely to be nonusers of tobacco (79.9%), living with children in the home (71.8%), at or above the poverty level (70.8%), non-white (76.0%), Hispanic (82.7%), and aged 45 or older (71.9%). The change in home tobacco use policies over two waves is shown in Table 2. Between Wave 3 (2014/2015) and Wave 4 (2016/2018), 11% of adults adopted a 100% ban on tobacco use in the home, but 8% of adults adopted weaker policies.

Table 3 presents the characteristics associated with adopting a home tobacco use policy between Wave 3 (2014/2015) and Wave 4 (2016/2018). Adults most likely to adopt a ban on combustible tobacco use in their homes were younger, non-Hispanic, tobacco users, and adults with no children living in the home.

### 5.2. Secondhand Smoke Exposure

The average number of hours of SHS exposure is shown in Table 4. Although the average number of hours that people are exposed to SHS is decreased overall, both adults and children continue to be exposed, either at home, in a car, at work, or outdoors. Among both adults and youths, those living in homes that completely banned all tobacco use for both survey waves assessed are exposed to SHS at a significantly lower rate. The highest level of SHS exposure is reported in households where tobacco use is allowed, and no home tobacco use policies are adopted. Living in a home that adopts a 100% tobacco-free home policy is associated with a 64% decreased SHS exposure among youths (a reduction of 5.3 average hours of exposure per week to 1.9 h) and a 69% decreased SHS exposure among adults (a reduction of 11.2 average hours of exposure per week to 3.6 h).

## 6. Discussion

This study describes trends in the prevalence of home tobacco use policies for three different types of tobacco products and their relationships with self-reported exposure to secondhand tobacco smoke. The estimates of SHS exposure are presented for varying levels of tobacco use, including smokers as well as non-smokers. We found:

- A total of 70% of adults completely ban the use of all types of tobacco, and 89% ban the use of at least one type of tobacco product inside their homes.
- Differences in policies were observed by tobacco use status and were consistent with previous research [21].
- Home tobacco use policies were more common among respondents living with children in the home, those at or above the poverty level, females, Hispanics, and non-whites, findings which were also shown previously in the scientific literature [22,23].

We found that home tobacco use policies are associated with lower levels of SHS exposure. Although our data were based on self-reported exposure to SHS, these findings were consistent with studies assessing cotinine, a marker of SHS, and showing declines in levels among both hospitality workers and the general public following the implementation of smoke-free laws [24,25,26]. Although the average number of hours that people are exposed to SHS has decreased, millions of children continue to be exposed in their home environments. We calculated that in the US, 10 million children are potentially exposed to combustible tobacco smoke inside their homes, 12 million are exposed to vaping emissions inside their homes, and 14 million are exposed to smokeless tobacco use. Youths who live in homes that allow all forms of tobacco use report an average of 8.0 h per week of exposure to secondhand smoke (data not shown), more than eight times as much as children who live in homes that do not allow any tobacco use.

The rate of tobacco-free home policy adoption is increasing, but progress is slow. Groups adopting 100% tobacco-free policies are younger tobacco users with no children in the home, suggesting a ceiling effect where the majority of adults have adopted home tobacco use policies, so the change we observe is in the harder-to-reach groups. Previous evidence suggests that some households face significant barriers to maintaining tobacco-free homes, including adverse weather, unpleasant or unsafe surroundings, and limited outside space [27], and our study found that 8% of adults report weaker home tobacco use policies over time. Therefore, opportunities exist for education and the continual reinforcement of tobacco-free home policies.

Interestingly, a sizeable minority of households ban one type of product (for example, combustible tobacco products) but allow another type (for example, ENDS). ENDS users perceive them as less harmful and report using them more frequently at home compared to cigarette users [28]. In contrast, the use of ENDS in public places is widely unrestricted [29]. Even fewer studies have examined home policies around the use of smokeless tobacco. Previous evidence suggests that smoke-free home rules may actually help prevent the uptake of smokeless tobacco [9,18], so it seems possible that policies around the use of smokeless tobacco would have a similar impact. Such findings call for investigators and clinicians to ask about home policies for each type of product, and this is an area that needs more study.

The strengths of this study include the use of a large, nationally representative sample and the assessment of multiple tobacco products. The limitations include the use of self-reported data, and the fact that estimates of SHS exposure were assessed as a whole from all exposure sources and were not restricted to SHS exposure in the home. The assessment of trends in cotinine levels in tobacco nonusers who live in homes where tobacco use is allowed would be an important next step to this work.

## 7. Conclusions

Most US adults have implemented tobacco-free home policies; however, there is still exposure to SHS, for both adults and children, particularly in the homes of tobacco users. Those who adopt completely tobacco-free homes experience a 2/3 reduction in hours of SHS exposure. These new data support the idea that health care professionals should ask about policies related to all tobacco use in the home, including the use of combustible tobacco, ENDS, and smokeless tobacco. Continued efforts regarding education about the importance of voluntary smoke-free rules in homes are warranted as part of a comprehensive approach to reducing tobacco use and SHS exposure.

## Figures and Tables

**Table 1:** Characteristics of adults with home tobacco use bans, PATH Study Wave 4: 2016–2018 (*n* = 27,755).

			All 3 Product Types Banned (*n* = 16,782)	Combustible-Tobacco Products Banned (Smokeless or ENDS Use Allowed, *n* = 4557)	Smokeless or ENDS Use Banned (Combustible-Tobacco Allowed, *n* = 2069)	No Tobacco Product Use Banned (*n* = 4347)	
Overall		%	69.5	14.5	5.3	10.6	
		95% CI	[68.5,70.5]	[13.8,15.3]	[5.0,5.8]	[10.0,11.2]	
Tobacco Use Status	No Past 30-Day tobacco use	%	79.9	12.0	3.4	4.7	**
		95% CI	[78.8,80.9]	[11.1,12.9]	[3.0,3.9]	[4.2,5.3]	
	Past 30-Day use of combustible-tobacco only	%	47.0	13.6	12.0	27.4	
		95% CI	[45.3,48.7]	[12.7,14.6]	[11.1,12.9]	[25.9,28.9]	
	Past 30-Day use of ENDS or smokeless tobacco only	%	28.9	52.3	5.9	12.8	
		95% CI	[25.8,32.3]	[49.0,55.6]	[4.5,7.7]	[11.2,14.7]	
	Past 30-Day poly use of combustible, ENDS, and/or smokeless tobacco	%	33.1	29.4	8	29.5	
		95% CI	[30.6,35.6]	[27.3,31.6]	[7.0,9.1]	[27.4,31.8]	
Children in the home (W1)	Yes	%	71.8	14.3	4.9	9.0	**
		95% CI	[70.4,73.1]	[13.3,15.3]	[4.4,5.5]	[8.4,9.8]	
	No	%	68.1	14.9	5.5	11.6	
		95% CI	[66.9,69.4]	[13.9,15.9]	[4.9,6.0]	[10.8,12.4]	
Poverty Status (W1)	Below poverty level	%	64.2	10.5	8.8	16.4	**
		95% CI	[62.4,66.0]	[9.7,11.5]	[8.1,9.6]	[15.2,17.8]	
	At or above poverty level	%	70.8	16.7	3.9	8.6	
		95% CI	[69.6,72.0]	[15.8,17.8]	[3.4,4.3]	[8.0,9.2]	
Sex	Male	%	67.4	15.7	5.1	11.9	**
		95% CI	[66.2,68.6]	[14.7,16.7]	[4.6,5.6]	[11.1,12.6]	
	Female	%	71.5	13.5	5.6	9.5	
		95% CI	[70.2,72.7]	[12.6,14.4]	[5.1,6.1]	[8.8,10.2]	
Race	White only	%	69.1	16.3	4.4	10.2	**
		95% CI	[67.9,70.2]	[15.4,17.3]	[4.0,4.8]	[9.6,10.9]	
	Black only	%	66.9	6.9	11.3	15.0	
		95% CI	[64.7,68.9]	[5.9,8.0]	[10.1,12.7]	[13.5,16.5]	
	other	%	76.0	10.5	5.5	8.1	
		95% CI	[73.4,78.3]	[8.8,12.4]	[4.3,6.9]	[6.9,9.6]	
Ethnicity	Hispanic	%	82.7	6.7	4.9	5.7	**
		95% CI	[81.1,84.2]	[5.7,7.8]	[4.3,5.6]	[5.0,6.5]	
	Not Hispanic	%	67.1	16	5.4	11.5	
		95% CI	[65.9,68.2]	[15.1,16.9]	[5.0,5.9]	[10.9,12.2]	
Age Group	18–24	%	65.4	15.3	6.6	12.6	**
		95% CI	[63.8,67.0]	[14.3,16.4]	[5.9,7.4]	[11.7,13.6]	
	25–44	%	67	18	4.9	10.2	
		95% CI	[65.5,68.5]	[16.9,19.1]	[4.4,5.4]	[9.4,11.0]	
	45+	%	71.9	12.3	5.3	10.4	
		95% CI	[70.6,73.2]	[11.3,13.3]	[4.7,6.0]	[9.7,11.2]	

Notes: ** *p* < 0.001 based on chi-square test. Estimates are from PATH Study public use data files weighted using cross-sectional (Wave 4 single-wave) weights. Home tobacco use bans were assessed with the questions: 1. For tobacco products that are burned, such as cigarettes, cigars, pipes or hookah, which statement best describes the rules about smoking a tobacco product inside your home? 2. Now think about other tobacco products that are not burned, like snus and other types of smokeless tobacco. Which statement best describes the rules about using these products inside your home? 3. Now think about e-cigarettes and other electronic nicotine products. Which statement best describes the rules about using these products inside your home? Response options were: “It is not allowed anywhere or at any time inside my home”, “It is allowed in some places or at some times inside my home” or “It is allowed anywhere and at any time inside my home”.

**Table 2:** Home tobacco use policy change between Wave 3 (2015/16) and Wave 4 (2016/18, *n* = 26,071).

	N	%	Lower	Upper
Complete ban on all tobacco use at both waves	11,639	58.5	57.4	59.6
Allow some tobacco use at baseline, all tobacco use banned at follow up	4115	11.1	10.5	11.6
Allow some tobacco use at both waves	7828	21.7	20.9	22.6
All tobacco use banned at baseline, allow some tobacco use at follow up	2489	8.7	8.2	9.2

Notes: Estimates are from PATH Study public use data files weighted using the longitudinal (all-wave) weights. Home tobacco use bans were assessed with the questions: 1. For tobacco products that are burned, such as cigarettes, cigars, pipes or hookah, which statement best describes the rules about smoking a tobacco product inside your home? 2. Now think about other tobacco products that are not burned, like snus and other types of smokeless tobacco. Which statement best describes the rules about using these products inside your home? 3. Now think about e-cigarettes and other electronic nicotine products. Which statement best describes the rules about using these products inside your home? Response options were: “It is not allowed anywhere or at any time inside my home”, “It is allowed in some places or at some times inside my home” or “It is allowed anywhere and at any time inside my home”. Results presented are inclusive of all tobacco use status categories.

**Table 3:** Characteristics of adults who adopt home tobacco policies between Wave 3 (2015/16) and Wave 4 (2016/18, *n* = 26,071).

					Odds	95% CI		
		N	%	95% CI	Ratio	Lower	Upper	
Overall		4115	11.1	[10.5,11.6]				
Tobacco Use Status	No Past 30-Day tobacco use	2313	10.1	[9.4,10.9]	1.00	Referent		
	Past 30-Day use of combustible tobacco only	1145	13.3	[12.5,14.1]	1.48	1.30	1.69	**
	Past 30-Day use of ENDS or smokeless tobacco only	224	13.6	[11.7,15.7]	1.34	1.03	1.75	*
	Past 30-Day poly use of combustible, ENDS, and/or smokeless tobacco	433	14.6	[13.0,16.3]	1.39	1.13	1.70	**
Children in the home (W1)	Yes	1010	9.1	[8.3,9.9]	1.00	Referent		
	No	1492	10.4	[9.7,11.2]	1.20	1.04	1.39	*
Poverty Status (W1)	Below poverty level	868	11.0	[10.2,11.9]	1.00	Referent		
	At or above poverty level	1413	9.3	[8.6,10.1]	0.88	0.78	1.00	
Sex	Male	2063	11.6	[10.9,12.3]	1.00	Referent		
	Female	2052	10.6	[9.8,11.4]	0.93	0.82	1.05	
Race	White only	2881	10.7	[10.1,11.3]	1.00	Referent		
	Black only	705	12.2	[11.2,13.3]	1.08	0.94	1.24	
	other	529	12.4	[10.7,14.3]	1.12	0.89	1.40	
Ethnicity	Hispanic	931	10.4	[9.3,11.6]	1.00	Referent		
	Not Hispanic	3184	11.2	[10.6,11.8]	1.24	1.03	1.49	*
Age Group	18–24	2075	21.4	[20.4,22.5]	1.4	1.2	1.7	**
	25–44	1095	10.1	[9.3,10.9]	1.1	1.0	1.3	
	45+	945	9.4	[8.5,10.3]	1.0	Referent		

Notes: * *p* < 0.05; ** *p* < 0.001. Estimates are from PATH Study public use data files weighted using the longitudinal (all-wave) weights. Home tobacco use bans were assessed with the questions: 1. For tobacco products that are burned, such as cigarettes, cigars, pipes or hookah, which statement best describes the rules about smoking a tobacco product inside your home? 2. Now think about other tobacco products that are not burned, like snus and other types of smokeless tobacco. Which statement best describes the rules about using these products inside your home? 3. Now think about e-cigarettes and other electronic nicotine products. Which statement best describes the rules about using these products inside your home? Response options were: “It is not allowed anywhere or at any time inside my home”, “It is allowed in some places or at some times inside my home" or "It is allowed anywhere and at any time inside my home”.

**Table 4:** Average hours of secondhand smoke (SHS) exposure in the past 7 days by home tobacco use policy change between survey waves.

	W1 (2013/14) to W2 (2014/15)		W2 (2014/15) to W3 (2015/16)		W3 (2015/16) to W4 (2016/18)		
	*n* = 22,547		*n* = 24,021		*n* = 25,806		
Adults	Hours SHS Exposure at follow up	95% CI	Hours SHS Exposure at follow up	95% CI	Hours SHS Exposure at follow up	95% CI	
Overall	5.0	[4.7–5.2]	4.6	[4.4–4.9]	4.2	[4.0–4.5]	*
Complete ban on all tobacco use at both waves	1.8	[1.7,2.0]	1.8	[1.6,2.0]	1.6	[1.5,1.8]	
Allow some tobacco use at baseline, all tobacco use banned at follow up	4.5	[4.0,5.0]	3.9	[3.4,4.4]	3.6	[3.1,4.0]	
Allow some tobacco use at both waves	13.3	[12.4,14.1]	12.8	[11.8,13.8]	11.2	[10.3,12.1]	*
All tobacco use banned at baseline, allow some tobacco use at follow up	6.8	[5.9,7.8]	5.0	[4.4,5.5]	5.5	[4.7,6.2]	
*p*-value (difference between policy change levels)	<0.001		<0.001		<0.001		
	W1 (2013/14) to W2 (2014/15)		W2 (2014/15) to W3 (2015/16)		W3 (2015/16) to W4 (2016/18)		
	*n* = 6880		8636		*n* = 10,197		
Youth (Based on parent self-report of home tobacco use bans)	Hours SHS Exposure at follow up	95% CI	Hours SHS Exposure at follow up	95% CI	Hours SHS Exposure at follow up	95% CI	
Overall	2.8	[2.5,3.2]	2.2	[1.9,2.5–2.7]	2.1	[1.9,2.4]	*
Complete ban on all tobacco use at both waves	1.4	[1.0,1.8]	1.1	[0.9,1.3]	0.9	[0.7,1.0]	
Allow some tobacco use at baseline, all tobacco use banned at follow up	1.9	[1.4,2.4]	1.4	[1.1,1.7]	1.9	[1.4,2.4]	
Allow some tobacco use at both waves	6.8	[5.7,7.9]	5.3	[4.4,6.1]	5.3	[4.6,6.0]	
All tobacco use banned at baseline, allow some tobacco use at follow up	2.0	[1.2,2.8]	2.6	[1.8,3.5]	2.9	[1.7,4.2]	
*p*-value (difference between policy levels)	0.10		<0.001		<0.001		

Notes: * Statistically significant change in mean hours SHS exposure. Means, and confidence intervals (CI’s) are from PATH Study public use data files weighted using longitudinal (all-waves) weights. Mean number of hours of SHS exposure was assessed with the question: “During the past seven days, about how many hours were you around others who were smoking [whether or not you were smoking yourself]? Include time in your home, in a car, at work, or outdoors”. Home tobacco use bans were assessed by asking, separately for combustible-tobacco/smokeless tobacco/ENDS, “Which statement best describes the rules about using tobacco products inside your home?” Response options were: “It is not allowed anywhere or at any time inside my home”, “It is allowed in some places or at some times inside my home” or “It is allowed anywhere and at any time inside my home”.

## Data Availability

The data presented in this study are openly available at https://doi.org/10.3886/ICPSR36498.v6 (accessed on 27 August 2021).
